# Krebs von den Lungen‐6 levels in untreated idiopathic pulmonary fibrosis

**DOI:** 10.1111/crj.13475

**Published:** 2022-01-26

**Authors:** Dingyuan Jiang, Huijuan Xiao, Run Dong, Jing Geng, Bingbing Xie, Yanhong Ren, Huaping Dai

**Affiliations:** ^1^ Department of Pulmonary and Critical Care Medicine, Center of Respiratory Medicine, China‐Japan Friendship Hospital National Clinical Research Center for Respiratory Disease, National Center for Respiratory Medicine Beijing China; ^2^ Department of Respiratory Medicine Capital Medical University Beijing China; ^3^ Department of Respiratory Medicine Zhengzhou Central Hospital Zhengzhou China

**Keywords:** diagnosis, idiopathic pulmonary fibrosis, KL‐6

## Abstract

**Background:**

Serum Krebs von den Lungen‐6 (KL‐6) has been reported to be elevated in patients with idiopathic pulmonary fibrosis (IPF).

**Objective:**

The aim of this study was to evaluate the diagnostic value of KL‐6 and whether the expression value of KL‐6 could indicate the severity of the disease in IPF patients. To address this question, it is necessary to see whether the patients' physical characteristics and other clinical conditions could affect the baseline KL‐6 level.

**Design:**

We conducted a study of 100 patients who were diagnosed with IPF. Lung function, computed tomography (CT), and serological lab tests data were analyzed.

**Results:**

The tests showed that there is a significant elevation of KL‐6 in IPF patients compared with other interstitial lung disease (ILD) and healthy controls. It was noted that serum KL‐6 is a stable biomarker not affected by lung infection and smoking, though IPF patients with antinuclear antibody (ANA) showed higher KL‐6 levels. KL‐6, in conjunction with poor pulmonary function and higher radiological fibrosis scores, indicates the severity of the disease but not poor survival.

**Conclusions:**

It is identified that serum KL‐6 is a useful noninvasive biomarker to help improve the certainty of IPF diagnosis from other interstitial lung disease and assist evaluation of disease severity and prognosis.

**Abbreviations**
ANA: antinuclear antibody
BALF: bronchoalveolar lavage fluid
CEA: carcino‐embryonic antigen
COP: cryptogenic organizing pneumonia
CTD: connective‐tissue disease
DLCO: diffusing capacity of the lung for carbon monoxide
ECLIA: electrochemiluminescence immunoassay
FEV1: forced expiratory volume in 1 s
FVC: forced vital capacity
HRCT: high‐resolution computed tomography
ILD: interstitial lung disease
IPF: idiopathic pulmonary fibrosis
KL‐6: Krebs von den Lungen‐6
ROC: receiver operating characteristic
TLC: total lung capacity

## 1 INTRODUCTION

Krebs von den Lungen‐6 (KL‐6) is a high‐molecular‐weight mucinous glycoprotein predominantly expressed on the alveolar type II cells in the lungs.^1^ KL‐6 is released increasingly when type II cells are involved in regenerating an injury. Serum KL‐6 levels are also found to increase in various diseases including interstitial lung disease (ILD) and some cancers, such as lung cancer and breast cancer.^2^, ^3^, ^4^ In fact, greater than 30% of patients with certain cancerous malignancies were positive for KL‐6.^5^ The cut‐off value of KL‐6 (500 U/ml) is well known as a diagnostic marker for distinguishing patients with ILD from healthy controls. However, the role of KL‐6 as a diagnostic marker in idiopathic pulmonary fibrosis (IPF) has not been thoroughly verified. Therefore, clarifying some clinical situations that could definitively affect KL‐6 levels is important.

IPF is a serious progressive disease. It is the most commonly seen disease among the ILD. Though specialists have discussed diagnosis criteria for several decades, no progress has been made in diagnostic tools used. High‐resolution computed tomography (HRCT) and bronchoscopy examination are still the most common methods in IPF diagnoses.^6^, ^7^ Today, rate and progression of the disease is still highly variable and unpredictable. Due to this unpredictability, it is helpful to see if there are noninvasive serological biomarkers that aid in determining the rate of IPF progression and in indicating the severity of the disease. Unfortunately, serum biomarkers are not yet mentioned in clinical guidelines as useful diagnostic tools for IPF.^8^, ^9^ This lack of mention is regrettable because KL‐6 has long been a well‐known biomarker for diagnosis and assessment for disease activity in ILD patients. One study showed that KL‐6 has a prognostic role for earlier acute exacerbation onset of IPF.^10^ Lamentably, though, another study showed degradation of KL‐6 during the progression of this disease.^11^ In addition, little research has been done to investigate what patients' clinical characteristics or laboratory examinations will do to serum KL‐6 levels. Because KL‐6 detection has been widely used in ILD, especially IPF patients, more research needs to be done to clarify the clinical significance of KL‐6.

In the study described below, we observed the serum and bronchoalveolar lavage fluid (BALF). The population sample used for this research was patients who were recently diagnosed with IPF and received no treatment before data collection. The aim of the study is to investigate the significance of KL‐6 in IPF patients and possible parameters that could affect KL‐6 levels.

## 2 METHODS

### 2.1 Study subject

All patients diagnosed with IPF from January 2007 to October 2014 who admitted to the Department of Pulmonary and Critical Care Medicine, Beijing Chao‐Yang Hospital, Capital Medical University were included in the IPF‐cohort study. Suspected IPF patients underwent standard investigation protocol based on the international diagnostic criteria for IPF.^6^, ^7^ Finally, 100 IPF patients were enrolled in this retrospective study, whereas 17 patients were excluded. Exclusion criteria included those who (1) took immunosuppressant or corticoid in the 6 months prior to enrollment and (2) lacked any clinical information or serum samples.

One‐hundred and twenty‐seven other‐ILD patients were enrolled. This group of patients includes 86 cryptogenic organizing pneumonia (COP) and 41 sarcoidosis patients. Two‐hundred and ninety‐eight healthy controls, which were the same age as the patients (±5 years), were chosen at the time of the health examination. The healthy controls had no history of any lung diseases and appeared normal chest X‐rays. We collected the clinical data, serological, and BALF samples at diagnosis. Clinical data included epidemiology information, physical examinations, HRCT, pulmonary function tests, and serological laboratory tests.

All the subjects enrolled in this study were given written consent to the detection of their serum or BALF samples for KL‐6 analysis, and anonymous use of their clinical records. The study was approved by the Capital Medical University Institutional Review Board (ethics approval no. 2016‐SSW‐10).

### 2.2 Serum and BALF measurements

Serum and BALF were taken at the time of diagnosis. Then, the samples were stored, until analysis, at −80°C after centrifugation. Serum KL‐6 levels were measured by sandwich‐type electrochemiluminescence immunoassay (ECLIA) using a Picolumi 8220 Analyzer (Eidia, Tokyo, Japan), as previously described.^12^

### 2.3 Statistical analysis

We calculated that a sample size of 97 patients would achieve the desired precision of the estimate of sensitivity of 90% and specificity of 80% at a two‐sided significance level of 0.05 with tolerance of 10%. Thus with the number of 100 IPF patients was sufficient to yield an exact lower 95% confidence interval.

All measurements were performed twice, and we expressed the data as the mean ± standard deviation (SD). A two sample *t* test was used to compare the continuous variables between groups. Comparison of non‐normally distributed variables between groups was done with the Mann–Whitney's *U* test. Furthermore, comparison of categorical variables was done with the chi‐square test. Correlation between two groups was analyzed using Pearson correlation analysis. Linear regression was used to examine the association between serum and IPF while adjusting for potential confounding variables. The receiver operating characteristic (ROC) curve was used to determine the optimal cut‐off values for serum biomarkers. The use of these cut‐off values allowed for the calculation of sensitivity, specificity, diagnostic accuracy, and discrimination of the IPF patients from the control subjects. The Grubb test was used to check for outliers. Normality of the data was verified using a histogram and a Q–Q plot. A *p* value of <0.05 is considered significant in the two‐tailed statistical test. All analyses were done using GraphPad Prism version 6 (GraphPad Software, San Diego, California, USA).

## 3 RESULTS

### 3.1 Patient characteristics

Details of the patients' characteristics are shown in Table 1. In the IPF group, more patients are smokers or ex‐smokers. Also, the smoking index, denoted by pack‐year, is higher. IPF patients are older than other‐ILD group patients.

**TABLE 1:** Characteristic of enrolled patients

	ALL (*n* = 525)	IPF (*n* = 100)	Other‐ILD (*n* = 127)	Health control (*n* = 298)
Age (years)+	57.4 (31–91)	65 (40–87)	52.2 (31–79)^*^	57 (37–91)
Gender, male/female	287/238	86/14	32/85	159/139
Smoker/ex‐smoker/nonsmoker	119/77/328	28/41/31	38/15/74	53/21/223^*^
Pack‐year+	24.4 (1.25–365)	30 (21–365)	30.5 (5–120)	20 (1.25–75)^*^
Duration of complaint to diagnose (month)	NA	39.13 (0.7–365)	5.5 (0.4–40)^*^	NA
Pulmonary function test				
FVC %pred	NA	74.57 ± 20.15	79.37 ± 21.8	NA
FEV1 %pred	NA	77.77 ± 25.83	75.5 ± 20.4	NA
TLC %pred	NA	71.15 ± 16.12	79.43 ± 19.8	NA
DLCO %pred	NA	39.71 ± 14.86	60.25 ± 20.5^*^	NA
Oxygenation index	NA	344.94 ± 112.08	375.7 ± 76.1	NA
HRCT score	NA	137.27 ± 35.1	NA	NA

*Note*: Parametric data are presented as means ± standard deviations; age and pack‐year are presented as medians (ranges).

Abbreviations: DLCO, diffusing capacity of the lung for carbon monoxide; FEV1, forced expiratory volume (first second); FVC, forced vital capacity; HRCT, high‐resolution computed tomography; TLC, total lung capacity.

* *p* < 0.05.

### 3.2 KL‐6 is significantly elevated in IPF patients

**FIGURE 1:**
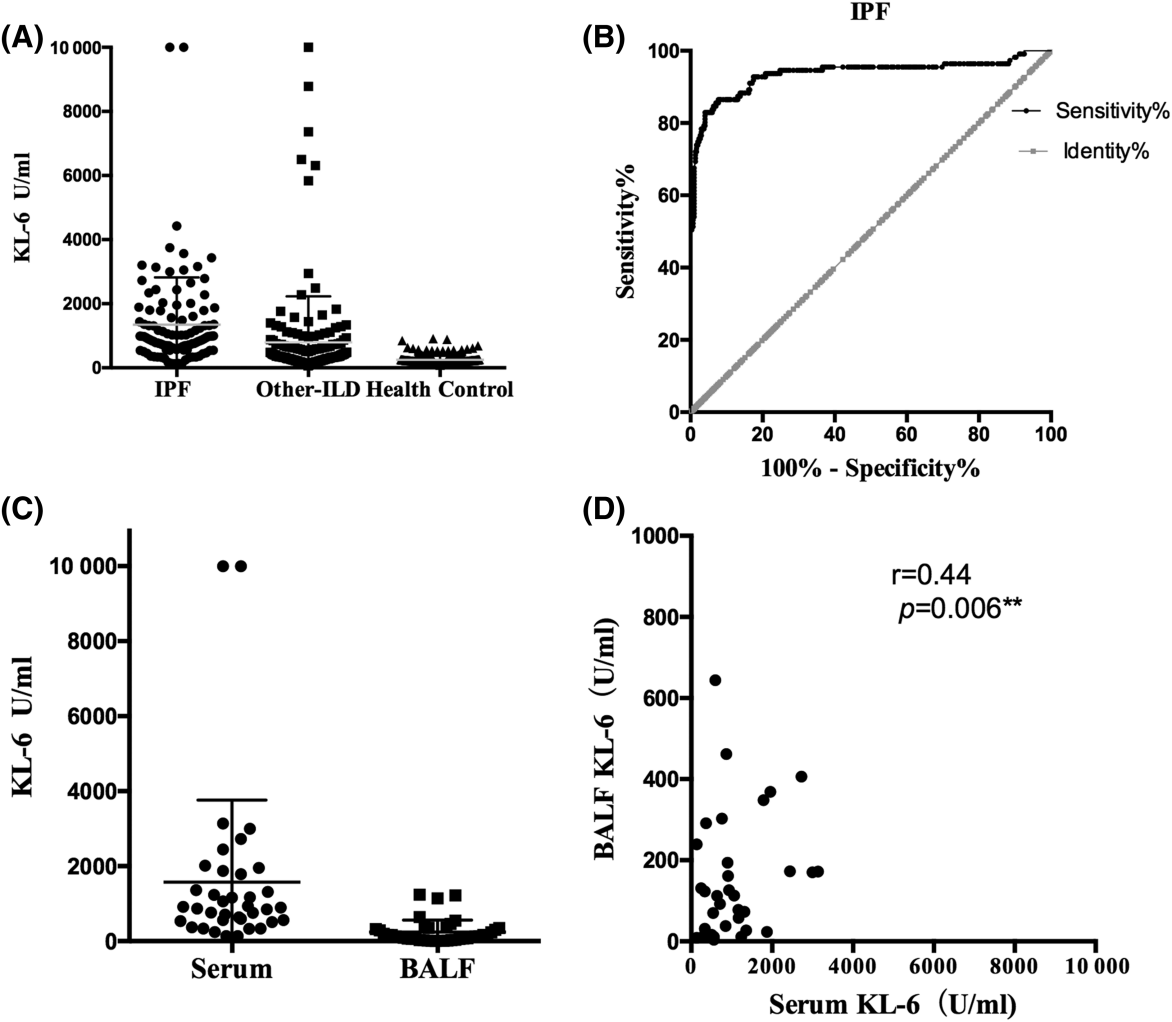
Serum and BALF KL‐6 levels and their diagnostic value. (A) Serum KL‐6 levels in IPF patients (*n* = 100), other‐ILD patients (*n* = 127), and healthy controls (*n* = 298). (B) Receiver operating characteristic (ROC) curve analysis of serum KL‐6. (C) Bronchoalveolar fluid KL‐6 levels in IPF patients (*n* = 41) is lower than that in serum. (D) The KL‐6 level in bronchoalveolar fluid and serum has a positive correlation with *r* = 0.4446, *p* < 0.01. BALF, bronchoalveolar lavage fluid; ILD, interstitial lung disease; IPF, idiopathic pulmonary fibrosis; KL‐6, Krebs von den Lungen‐6

We detected the KL‐6 concentration in periphery blood serum. KL‐6 levels are significantly increased in IPF patients (1344 ± 140.4 U/ml) compared with both other‐ILD patients (850.7 ± 125.4 U/ml) and healthy controls (248.6 ± 7.406 U/ml) (Figure 1A). The KL‐6 ROC curve in IPF is shown in Figure 1B. The KL‐6 cut‐off value is 540.5 U/ml with a sensitivity of 81.98% and a specificity of 95.97%. Also, the likelihood ratio is 20.36 (95% confidence interval [CI] 0.90 to 0.97, *p* < 0.0001). Given this data, one can see that KL‐6 performs well as a valuable diagnostic marker in IPF.

### 3.3 Serological KL‐6 level is higher than KL‐6 levels in BALF

In total, there were 41 IPF patients in this research from whom BALF was drawn. After filtration and centrifugation, we detected KL‐6 levels in BALF supernatants using the same protocol as in serum KL‐6 analysis. KL‐6 levels in serum were significantly higher than in the lavage fluid (1344 ± 140.4 U/ml vs. 245.2 ± 49.1 U/ml, *p* < 0.001) (Figure 1C). However, there is a positive correlation between KL‐6 in serum and in BALF (*r* = 0.4446, *p* < 0.01) (Figure 1D). The lower KL‐6 level in BALF is most likely due to the low level of total protein concentration. Therefore, the data suggest the noninvasive periphery blood could reveal inner lung KL‐6 levels.

### 3.4 Higher serum KL‐6 levels present more serious disease

The survival status of 33 IPF patients was obtained by the end of year 2019. Twenty‐six patients died. The survival time of these 26 patients are 16.5 months after diagnosis. The minimum and maximum of survival time are 4 and 63 months. The correlation between KL‐6 value and survival time has no significant differences (*r* = −0.23, *p* = 0.26).

Pulmonary function test, HRCT fibrosis score assessed based on previous report by Oda et al,^13^ and the arterial blood oxygenation index are excellent indicators to evaluate patients' lung function impairments. All of the IPF patients were separated into three groups based on their serological KL‐6 levels. There were no obvious differences in the age or gender between different groups. Likewise, no significant difference was seen in the oxygenation index among different groups. However, patient groups with lower levels of KL‐6 (KL‐6 < 500 U/ml) tended to have less proportion of nonsmokers. Additionally, patients with serum KL‐6 levels greater than 1000 U/ml had significantly worse lung function values in forced vital capacity (FVC) %pred, forced expiratory volume in 1 s (FEV1) %pred, and total lung capacity (TLC) %pred and higher radiological fibrosis scores compared with the group with serum KL‐6 < 500 U/ml. Patients with serum KL‐6 levels between and 1000 U/ml had a tendency for a worse FVC %pred level than those KL‐6 < 500 U/ml. However, the lung function results between groups with KL‐6 > 1000 U/ml and 500 U/ml < KL‐6 < 1000 U/mL did not show significant differences (Table 2).

**TABLE 2:** Characterizations of patients with different levels of KL‐6

	KL‐6 < 500 U/ml (*n* = 17)	500 U/ml < KL‐6 < 1000 U/ml (*n* = 43)	KL‐6 > 1000 U/ml (*n* = 40)	*p*1	*p*2	*p*3
Age (years)+	65(44–81)	66(40–87)	66(41–87)	0.91	1.67	0.75
Gender, male/female	13/4	71/12	32/8	0.46	0.73	0.44
Smoker/ex‐smoker/nonsmoker	7/8/2	31/33/19	8/15/17	0.58	0.058	0.046*
FVC %pred	83.8 ± 16.2	73.0 ± 20.5	67.3 ± 21.0	0.056	0.005**	0.21
FEV1 %pred	85.6 ± 17.7	76.5 ± 20.2	71.3 ± 21.9	0.11	0.018*	0.23
TLC %pred	77.5 ± 15.9	70.4 ± 16.3	67.5 ± 11.2	0.13	0.012*	0.44
DLCO %pred	40.5 ± 15.0	40.1 ± 14.7	35.3 ± 13.4	0.92	0.17	0.1
Oxygenation index	342.0 ± 113.5	349.7 ± 118.0	341.33 ± 127.81	0.81	0.97	0.74
HRCT fibrosis score	134 ± 34.3	137.6 ± 33.7	151.8 ± 41.2	0.73	0.013*	0.22

*Note*: Parametric data are represented as means ± standard deviations; ages are represented as median (range). *p*1 represents a *p* value between KL‐6 < 500 U/ml and 500 < KL‐6 < 1000 U/ml; *p*2 represents a *p* value between KL‐6 < 500 U/ml and KL‐6 > 1000 U/ml; *p*3 represents a *p* value between KL‐6 > 1000 U/ml and 500 < KL‐6 < 1000 U/ml.

### 3.5 Patients with ANA positive have higher serum KL‐6 levels

**FIGURE 2:**
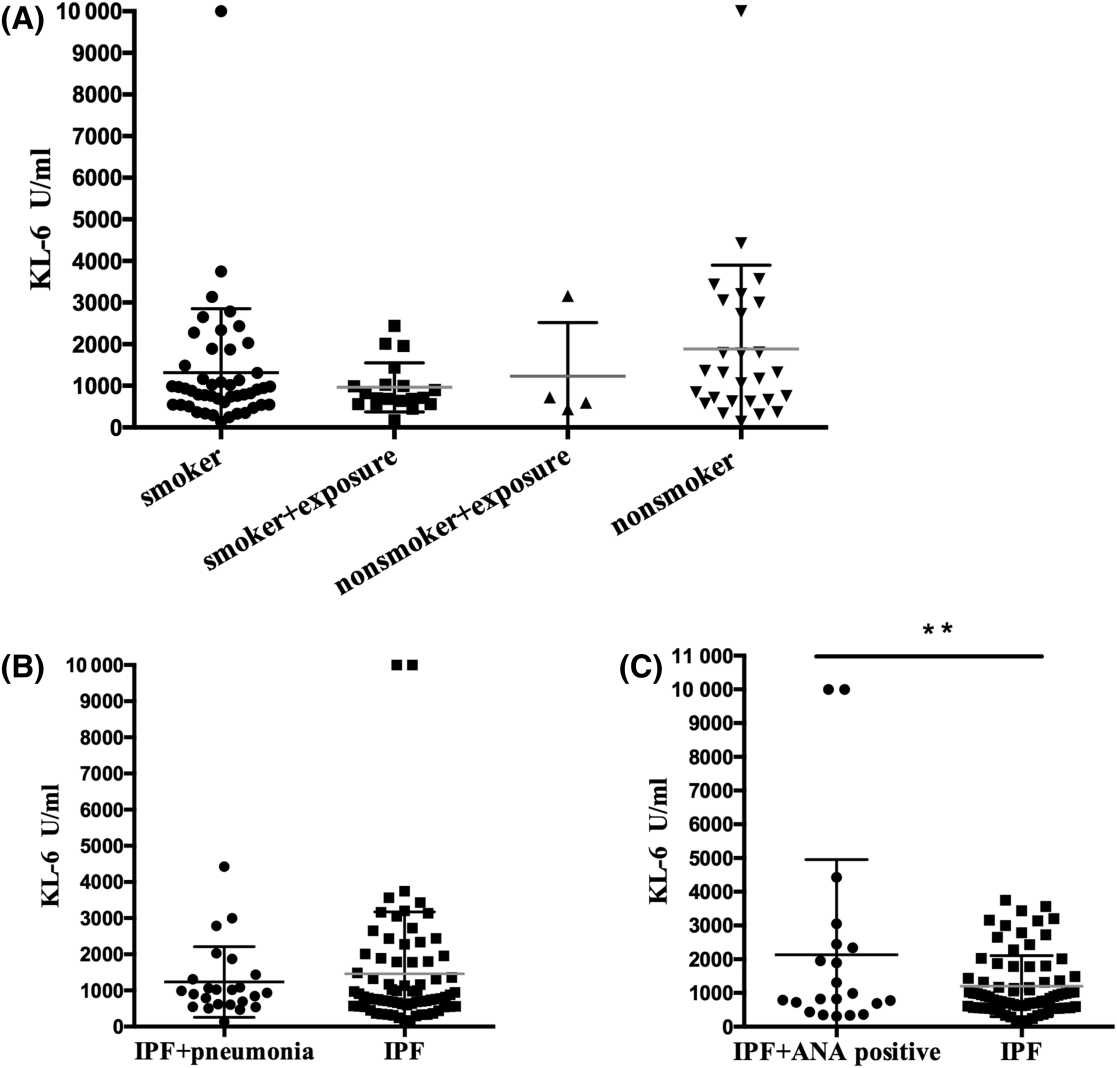
Serum KL‐6 levels in relation to different clinical characteristics. (A) Serum KL‐6 levels in IPF patients who have a history of smoking (*n* = 50, KL‐6 1318 ± 1534 U/ml), smoking history with environmental exposure (*n* = 19, KL‐6 961 ± 591 U/ml), nonsmoker with environmental exposure (*n* = 4, 1229 ± 1292 U/ml), and nonsmokers (*n* = 27, 1887 ± 2011 U/ml). (B) Serum KL‐6 levels in IPF patients who have pneumonia (*n* = 24, 1234 ± 199.2 U/ml) and who do not have pneumonia (*n* = 76, 1459 ± 201.0 U/ml). (C) IPF patients who were serum antinuclear antibody positive (*n* = 21) in conjunction with a higher serum KL‐6 level (2226 ± 639.8 U/ml vs. 1201 ± 104.1 U/ml, *p* < 0.01). ANA, antinuclear antibody; ILD, interstitial lung disease; IPF, idiopathic pulmonary fibrosis; KL‐6, Krebs von den Lungen‐6

We investigated whether some comorbidities or positive serological laboratory tests in IPF patients would affect KL‐6 levels. Because KL‐6 reflects epithelium injury, we considered whether smoking or long‐time environmental exposure (to any inorganic or organic substance) may have an effect on KL‐6 secretion. Sixty‐nine patients (69%) were current smokers or ex‐smokers with an average of pack‐year of 39.07. Twenty‐three patients (23%) had long‐term exposure due to their work or household environment. Among the environmental exposure patients, four patients were exposed to animals, three were exposed to coal, three were exposed to dust, three were exposed to steel, two were exposed to asbestos, and two were exposed silica. Moreover, the remaining seven were exposed to organic substances via benzene, plastic, diamonds, paint, formaldehyde, wood, or chlorine respectively. The KL‐6 levels did not show significant differences between people who had smoked or had environmental exposure (Figure 2A).

Pulmonary infection is one of the most commonly happened comorbidity in IPF patients; therefore, we analyzed patients to see if pneumonia affects KL‐6 levels. Twenty‐four patients (24%) were diagnosed with pneumonia according to community acquired pneumonia diagnosis criteria at the time of serum collection.^14^ In addition, microbe culture from sputum or BALF confirmed four patients had *Candida albicans*, one had Tropical candida, one had Klebsiella pneumonia, one had Haemophilus influenza, and one had Streptococcus pneumonia. No differences were seen in patients with or without pneumonia (Figure 2B).

Twenty‐one (21%) patients had a low titer antinuclear antibody (ANA) in serum, but they did not have any clinical domain or meet any criteria of connective‐tissue disease (CTD). Interestingly, patients that tested positive for ANA antibody (3 with 1:320 and 18 with 1:100) had higher KL‐6 expression compared with those without ANA antibody (Figure 2C). Clinical characteristics of patients with or without ANA antibody were analyzed. No significant difference between the patients in regard to lung functions or cell classification in lavage fluids (Table 3). This result suggests an ANA antibody in blood may influence KL‐6 secretion.

**TABLE 3:** Characteristics of IPF patients with ANA positive

	ANA positive (*n* = 21)	ANA negative (*n* = 79)	*p*
Age (years)+	67(55–84)	64.5(40–87)	0.59
Gender, male/female	16/5	69/10	0.29
KL‐6 level (U/ml)	2226 ± 639.8	1201 ± 104.1	0.009**
FVC %pred	74.4 ± 3.0	74.7 ± 5.8	0.96
FEV1 %pred	79.0 ± 5.7	77.3 ± 3.0	0.78
TLC %pred	67.0 ± 5.8	72.4 ± 2.2	0.29
DLCO %pred	34.5 ± 3.9	41.6 ± 2.5	0.16
BALF classification	*n* = 13	*n* = 40	
Lymphocyte (%)	7.8 ± 1.5	6.4 ± 0.8	0.4
Neutrophil (%)	37.3 ± 5.6	46.6 ± 3.8	0.22
Macrophage (%)	52.0 ± 6.0	44.7 ± 3.5	0.3

*Note*: Parametric data are represented as means ± standard deviations; ages are represented as median (range). BALF classifications are represented as frequency and percentage.

** *p* < 0.01.

### 3.6 KL‐6 level has a correlation with CEA level and BALF lymphocytes

**FIGURE 3:**
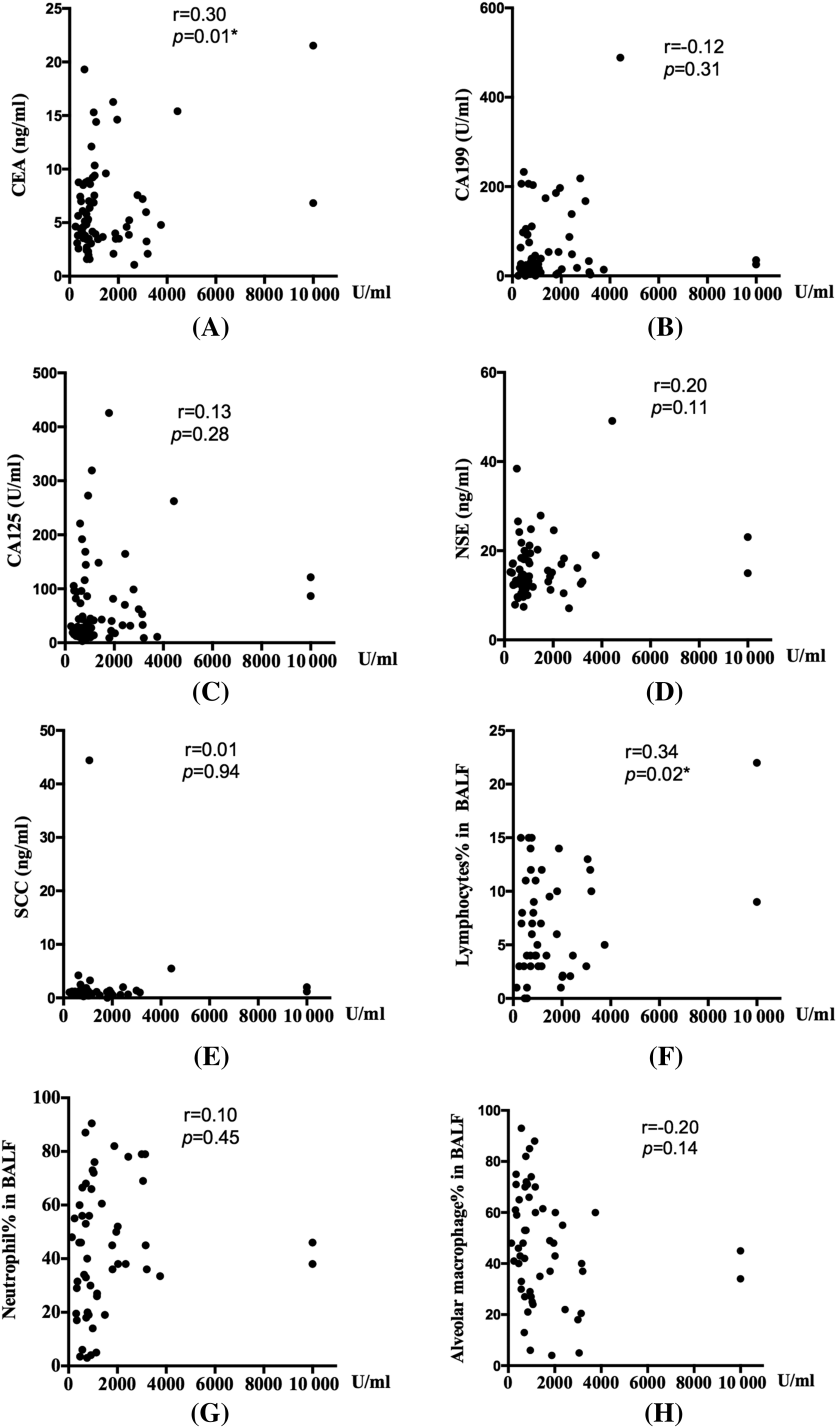
Correlations between serum KL‐6 levels and tumor biomarkers such as (A) CEA, (B) CA199, (C) CA125, (D) NSE, and (e) SCC; as well as BALF cell proportions: (F) lymphocytes, (G) neutrophils, and (H) alveolar macrophage. BALF, bronchoalveolar lavage fluid; CEA, carcino‐embryonic antigen; NSE, neuron‐specific enolase; SCC, squamous cell carcinoma antigen

KL‐6 could increase in patients with lung cancers. No IPF patient enrolled in this research had cancer. We analyzed the relations between KL‐6 and other commonly used tumor biomarkers. Among all the data, KL‐6 shows a weak positive correlation with carcino‐embryonic antigen (CEA) levels (*r* = 0.3055, *p* < 0.05). Cell classification in BALF is a useful tool in ILD diagnosis and somehow could predict the reaction to treatment. Cell count and classification through cell smear sections were processed as soon as we obtained bronchoalveolar lavage. BALF cell classification was performed by two independent specialists. KL‐6 levels in the blood have a weak positive correlation with the proportion of lymphocytes in the whole BALF cells (*r* = 0.3436, *p* < 0.05) (Figure 3).

## 4 DISCUSSION

In the present study, it has been shown that IPF patients have increased serological KL‐6 levels compared with healthy controls as well as other‐ILD patients. The value of KL‐6 in BALF is significantly lower than that in serum, however, with a positive correlation between them. Patients with higher KL‐6 levels, especially higher than 1000 U/ml, tend to show worse lung function. Interestingly, patients who had a low titer of ANA show higher KL‐6 levels in serum. Whether smoke or expose to other environmental pollute did not show differences in serological KL‐6 values. These results suggest that KL‐6 is an effective diagnostic marker for IPF.

KL‐6, a high‐molecular‐weight glycoprotein classified as “cluster 9” (MUC‐associated), is supposed to indicate numbers of regenerating type II pneumocytes. Purified KL‐6 is also a chemoattractant for fibroblasts.^15^ More and more studies suggest KL‐6 play significant role in ILD diagnosis and disease evaluation. Okamoto et al^16^ examined KL‐6 levels in hypersensitivity pneumonitis (HP) and IPF patients and found that KL‐6 value is much higher in HP patients. Especially in those patients with CTD, higher expression of KL‐6 indicates pulmonary involvement.^17^ Sometimes, the clinical features of chronic sarcoidosis, organizing pneumonia, and IPF are similar. In this study, we examined KL‐6 in IPF patients, COP, and sarcoidosis patients. IPF patients appear to have higher KL‐6 levels than COP and sarcoidosis. Both of these patients have higher KL‐6 levels than healthy controls. It suggests that KL‐6 is a good diagnostic marker for pulmonary impairment and IPF patients tend to have higher KL‐6 value. We suggested KL‐6 could assist diagnose IPF especially when a differential diagnosis is required in several ILD.

Researchers also conduct a lot of studies to show the prognostic value of KL‐6. KL‐6 could be a useful marker for monitoring the therapeutic effect of drugs as it continues to elevate in patients who have died.^18^ Changes in KL‐6 levels were significantly inversely correlated with changes in percentages of FEV1, TLC, diffusing capacity of the lung for carbon monoxide (DLCO), and residual volume in polymyositis/dermatomyositis patients with ILD.^19^, ^20^ In our study, higher KL‐6 also showed connection with lower FVC, FEV1, and DLCO level in IPF patients. Wakamatsu et al^21^ found that IPF patients with increased serum KL‐6 levels during follow‐up had steeper decline in FVC. Patients with initial higher KL‐6 level and increased KL‐6 during follow‐up tend to have poor survival. In this study, we confirmed similar result that a basal level of higher KL‐6 indicates worse lung function at that moment. However, initial KL‐6 levels did not show correlation with survival time in this study, and taken together with both lung function and HRCT scoring confirmed KL‐6 represents with more severe disease. KL‐6 value varies with disease progress. The protein's role as a prognostic biomarker is controversial as several studies have had different conclusions. Higher KL‐6 levels were associated with a higher risk of developing acute exacerbation as well as 3‐month mortality in IPF patients.^22^ Wakamatsu et al^21^ conducted a retrospective study of IPF patients and found a naturally decline of KL‐6 also in association with disease progression. Some of the patients enrolled in this study had long time of respiratory complain but no visit to the doctor ever before, they had shorter survival time and lower FVC, with not that high KL‐6 value. KL‐6 level in IPF patients with different progression needs more prospective studies and more follow‐up information.

Serum KL‐6 levels could be affected by patients' age, ethnicity, and polymorphisms in the MUC1 gene, as indicated before.^23^ Few studies have been done on the effects of other serological tests on KL‐6 expression. We found no significant variations between patients' who had smoking history or environmental exposure. The additional caveat of pulmonary infection did not affect serum KL‐6 levels either. KL‐6 in serum and BALF had significantly correlation. These data suggest that KL‐6 is kind of stable biomarker and we can examine its value in serum instead of in the BALF. Impressively, patients with a low titer of serum ANA levels have significantly higher serum KL‐6 levels. This result is never reported before as far as we know. Antinuclear antibodies are autoantibodies bound to structures within the cell nucleus.^24^ Positive titers of less than 1:160 are present in up to 20% of the healthy population, especially the elderly. A low titer of ANA positive was found in healthy people and people that were in autoimmune disease remission.^25^, ^26^ Of all the IPF patients enrolled in this study, no patients showed any CTD characteristics at the time of diagnosis. We concluded that these patients conform to IPF criteria. As indicated before, KL‐6 elevated in CTD‐related ILD. KL‐6 elevation not only suggests pulmonary involvement but also indicates poor prognosis.^27^, ^28^ KL‐6 levels with cut‐off value of 1273 U/ml were predictive of end‐stage lung disease in systemic scleroderma‐ILD. Furthermore, patients with CTD without ILD do not have significantly higher KL‐6 levels compared with controls.^29^, ^30^ Previous studies suggest KL‐6 as domain related specifically to lungs in CTD patients. The pathogenesis of many connective‐tissue diseases involve mainly T cell immunity disorders, and the inflammatory cytokines exerted by T‐lymphocytes and autoantigens can cause alveolar epithelial cell injury. This is one of the reasons for elevated KL‐6 secretion. ANA as an important autoimmune antibody may also play a role in epithelial cell injury. This maybe one of the reasons that ANA‐positive patients had higher KL‐6 value. Autoantibodies play roles in the pathogenesis of pulmonary fibrosis, but the mechanisms and specific correlations between autoantibodies and pulmonary fibrosis are not yet well defined yet. Though the lung function test did not show significant differences between patients with or without ANA positive, the average DLCO and TLC levels are lower in ANA‐positive group. Hence, we need more studies on ANA‐positive patients to clarify the relations of KL‐6 and IPF in patients.

KL‐6 that plays as a prognostic biomarker is also controversial. In this study, we confirmed that serum KL‐6 was higher in BALF especially in IPF patients when compare with cryptogenic organized pneumonia and sarcoidosis patients. KL‐6 was also shown to be a stable marker, which could not be affected by lung infection or previous smoking history, which is commonly seen in ILD patients. These results suggested that noninvasive KL‐6 performed as a diagnostic biomarker in IPF. What's more, this study collected not only the lung function but also HRCT fibrosis scoring data to evaluate the role of KL‐6 in disease severity. This study gave a comprehensive study of initial KL‐6 in IPF diagnosis and evaluation, and it was shown that KL‐6 was insufficient to assess prognosis.

## CONFLICT OF INTEREST

The authors declare no conflict of interest.

## ETHICS STATEMENT

All the subjects enrolled in this study were given written consent to the detection of their serum or BALF samples for KL‐6 analysis, and anonymous use of their clinical records. The study was approved by the Capital Medical University Institutional Review Board (ethics approval no. 2016‐SSW‐10).

## AUTHOR CONTRIBUTIONS

DJ and HD designed the study; DJ, HX, RD, and JG performed the experiments and/or analyzed the data; DJ, BX, and RY collected the clinical data; DJ and HD wrote the manuscript.

## Data Availability

I confirm that my Data Availability Statement (pasted below) complies with the Expects Data Policy.
